# Proceedings of Dental Associations

**Published:** 1855-10

**Authors:** 


### ARTICLE XIV.

### PROCEEDINGS OF DENTAL ASSOCIATIONS.

### *Pennsylvania Dental Association.*

At the annual meeting of the Pennsylvania Association of Dental Surgeons, held on Tuesday evening, October 3d, 1854, the following officers were elected to serve for the ensuing year.

*President*—Daniel Neall, D. D. S.; *Vice-President*—Elisha Townsend, M. D., D. D. S.; *Recording Secretary*—Chas. A. Du Bouchet, M. D., D. D. S.; *Treasurer*—F. Reinstein, D. D. S.; *Librarian*—James M. Harris, M. D., D. D. S.; *Examining Committee*—Edward Townsend, D. D. S., J. H. M'Quillen, M. D., D. D. S., Chas. A. Du Bouchet, M. D., D. D. S., Wm. Fouchè, D. D. S., David Roberts, D. D. S.

Among other subjects of importance disposed of at this meeting, the following code of ethics, previously discussed, was adopted and ordered to be printed for distribution.

CHAPTER I.—*Of the Duties of Dentists to their Patients, and of the Obligations of Patients to their Dentists.*

ARTICLE I.—*Duties of Dentists to their Patients.*—*Sec.* 1. A dentist should not only be ever ready to keep his professional engagements, but his mind ought always to be imbued with the importance of his function, and the responsibility he habitually incurs in its discharge. These obligations are the more deep and enduring, because there is no tribunal, other than his own conscience, to adjudge penalties for his carelessness or negligence. Dentists should therefore be scrupulously loyal to their trust, reflecting, that the ease, health, and no inconsiderable share of the happiness of those committed to their charge, depend on their skill, attention and fidelity. They should study also, in their deportment, so to unite tenderness with firmness, and condescension with authority, as to inspire the minds of their patients with gratitude, respect and confidence

*Sec.* 2. Every case committed to the charge of a dentist should be treated with attention, steadiness and humanity. Reasonable indulgence should be granted to the mental peculiarities and caprices of his patient. Secrecy and delicacy, when required by peculiar circumstances, should be strictly observed; and the familiar and confidential intercourse to which dentists are admitted, in their professional relations, should be used with discretion, and the most scrupulous regard to fidelity and honor. The obligation of secrecy extends beyond the period of professional services; none of the privacies of personal or domestic life, no infirmity of disposition or flaw of character, observed during professional attendance, should ever be divulged by him, except when he is imperatively required to do so. The force and necessity of this obligation are indeed so great, that professional men have, under certain circumstances, been protected in their observance of secrecy by courts of justice.

*Sec.* 3. A dentist ought equally to avoid unfavorable prognostications and boastful promises, because they savor of empiricism, by magnifying the importance of his services in the treatment of the case, and abusing the simple confidence of those who best deserve his sincerity. It is therefore a sacred duty to guard himself carefully in these respects, and to avoid all things, in word, action or manner, which have a tendency to bias the judgment or impose upon the feelings of his patient.

*Sec.* 4. Consultations should be favored in difficult and doubtful cases, as they give rise to confidence, energy and more enlarged views of practice, and eminently tend to liberalize the professional relations of practitioners

*Sec.* 5. Hygienic counsel within the proper province of the dentist, concerning as it does those personal habits which are so prevalent and so offensive in the social life of our country, should be urged the more earnestly as rules of health, for the reason that they promote the minor morals of the patient. Advice, strictly professional, is never impertinent, and is generally authoritative and acceptable in proportion to its respectful boldness.

Article II. *Obligations of Patients to Dentists.—Sec.* 1. The members of the dental profession, upon whom is enjoined the performance of duties so important as theirs are to the community, and from whom are required the devotion of their best efforts for the welfare of those who require their services, have a right to expect that their patients should entertain a just sense of their reciprocal obligations.

*Sec.* 2. The first duty of a patient is to select, as his dentist, one who has received a regular professional education. In no trade or occupation do men rely upon the skill of an untaught artist; and in dental surgery, confessedly one of the most difficult and intricate of the arts, the world ought not to suppose that knowledge is intuitive.

*Sec.* 3. Patients should prefer a dentist whose habits of life are regular, and who is not devoted to company, pleasure, or to any pursuit incompatible with his professional studies and duties. A patient should also confide the care of himself and family,as much as possible to one dentist, for a practitioner who has become acquainted with their peculiarities of constitution, is more likely to be successful in his practice than one who does not possess such knowledge. A patient should apply for advice in the early stage of disease, and in what may appear to him trivial cases, that the remedial treatment may be completely successful; and as incipient diseases of the dental system are open to very early detection by proficients in the science, so protective and preventive treatment is even more certain and available, than in any other branch of remedial medicine, and should be the more carefully secured.

*Sec.* 4. The obedience of a patient to the prescriptions of his dentist, should be prompt and implicit. He should never permit his own crude opinions to influence his attention to them, or to excuse his negligence. Preventive and remedial measures are based upon physiological principles, whose agency is not always in apparent correspondence with the means employed to make them available.

*Sec.* 5. Patients should always, when practicable, call upon their dentist within the hours appropriated to consultation; they should keep their appointments with rigid punctuality; and they should respect his necessary times of refreshment, recreation and repose. A full practice is so laborious and exhausting, and interference with engagements so injurious, that a careful consideration for them, ranks among the highest duties of the patient to the practitioner.

*Sec.* 6. The unfavorable criticisms of friends and physicians, upon the operations of the dentist, ought to be submitted to him for his consideration and explanation, before they are allowed any decisive influence upon the mind or conduct of the patient.

Chapter II.—Article I.—*Duties for the Support of Professional Character.—Sec.* 1. Every individual, on entering the profession, as he becomes thereby entitled to its privileges and immunities, incurs an obligation to exert his best abilities to maintain its dignity and honor, to exalt its standing, and to extend the bounds of its usefulness. He should, therefore, observe strictly such laws as are instituted for the government of its members; should avoid all contumelious and sarcastic remarks relative to the faculty as a body; and while, by unwearied diligence, he resorts to every honorable means of enriching the science, he should entertain a due respect for his seniors, who have by their labors brought it to the advanced condition in which he finds it.

*Sec.* 2. It is considered derogatory to the dignity of the profession to resort to public announcements, or private cards or handbills, inviting the attention of the public to particular methods of treatment; publicly to offer gratuitous advice, or to promise radical or extraordinary cures; or to publish cases and operations in the public newspapers, or suffer such publications to be made; to adduce certificates of skill and success, or to boast of cures and remedies. These are the ordinary practices of empirics, and are highly reprehensible in a regular dentist.

ARTICLE II.—*Professional Services of Dentists to each other.* Dental practitioners, their wives and children, have no just claim upon other practitioners for gratuitous services. They may be accorded, for such reasons as arise out of the relation and condition of the parties as individuals, but from the nature of such services they cannot properly be demanded as a comity of the profession.

ARTICLE III.—*Of the Duties of Dentists in regard to Consultation.—Sec.* 1. A regular dental education furnishes the only presumptive evidence of professional abilities and acquirements, and ought to be the only acknowledged right of an individual to the exercise and honors of the profession. Nevertheless, no intelligent regular practitioner, who is of good moral and professional standing in the place in which he resides, should be fastidiously excluded from professional fellowship, nor should his aid be refused in consultation, when it is desired by the patient. But no one can be considered as a regular practitioner, or a fit associate in consultation, who, in his practice, rejects the accumulated experience of the profession, and the aids furnished by anatomy, physiology, pathology and organic chemistry.

*Sec.* 2. In consultation, no rivalship or jealousy should be indulged; candor, probity, and all due respect should be exercised towards the dentist having charge of the case.

*Sec.* 3. A dentist who is called upon to consult, should observe the most honorable and scrupulous regard for the character and standing of the practitioner in attendance: the practice of the latter, if necessary, should be justified as far as it can be, consistently with a conscientious regard for truth, and no hint or insinuation should be thrown out which could impair the confidence reposed in him, or affect his reputation. The consulting dentist should also carefully refrain from any of those extraordinary attentions or assiduities, which are too often practiced by the dishonest, for the base purpose of gaining applause or ingratiating themselves into the favor of families and individuals.

*Sec.* 4. When a dentist is consulted by a patient of another practitioner, in consequence of the absence or sickness of the latter, he ought to limit his interference to the necessary treatment demanded by the case; and when it has been only incidental, and not requiring his own continued attention, on return or recovery of the regular attendant, with the consent of the patient, he ought to surrender the case.

The following gentlemen were unanimously elected active members: Wm. Calvert, D. D. S.; Wm. Gorgas, D. D. S., Philadelphia; Thos. A. Shaw, D. D. S., Alabama. And in view of the services rendered to the dental profession by Dr. John R. McCurdy, he was, also, unanimously elected an honorary member of the Association.

At the stated meeting of the Pennsylvania Association of Dental Surgeons, held on December 5th, 1854, the principal business was the discussion at large of the report of the Committee on Anæsthetic agents; the subject was considered of such importance as to require another meeting expressly to further discuss this topic, and consequently an adjourned stated meeting was held on Tuesday, December the 12th, when after a thorough debate, the report of the Committee on Anæsthetics was adopted, and ordered to be published.—*Dental News Letter.*

### *Eleventh Annual Meeting of the Mississippi Valley Association of Dental Surgery.*

SOCIETY met pursuant to adjournment at 10 o'clock, Wednesday, February 21, 1855, in the lecture room of the Ohio College of Dental Surgery. The President, Dr. Goddard, in the chair.

Present, Drs. Taft, Taylor, Bonsal, Ulrey, Talbot, Watt, Smith, Webster, and Leslie.

On motion, Drs. Ulrey and Watt were appointed on the executive committee, in place of absent members. After an absence, this committee presented the following report, which was adopted. The executive committee respectfully submit the following report, as the order of business.

I. Report of the examining committee.

II. " of officers.

III. " of standing committees.

IV. Addresses and reading of essays.

V. Miscellaneous business.

1st. What is the best means to allay the sensibility in teeth in which the nerves are not actually exposed?

2d. Is any non-conductor to be used previous to filling, if so, what is best?

3d. How should teeth be treated in case the nerves are exposed?

4th. What is the best method of destroying the pulp of a tooth?

5th. What is the best material for filling teeth, and how should it be prepared?

6th. Has risodontropy borne the test of experience?

7th. What is the best material for hard casts?

8th. Do either Allen's or Hunter's continuous gums, bear the test of mastication as well as teeth on ordinary plate or block work?

9th. What is the experience of the profession in regard to block tin, india rubber, or gutta percha bases for either temporary or permanent sets of teeth, and what is the best method of manipulation in their preparation?

10th. What is the experience of the profession in the use of chloroform and ether, in regard to mental hallucinations?

11th. How does the material known as Hill's stopping, answer as a temporary filling, and is at all affected by the fluids of the mouth?

12th. What is the best method of inserting plates by air pressure?

A. S. Talbert,
George Watt,
J. P. Ulrey.

The examining committee, presented the name of W. H. Howe, D. D. S., of Cincinnati, for membership. On balloting, Dr. Howe was declared unanimously elected.

The treasurer, presented the following report which was accepted.

*To the M. V. A. of Dental Surgery.*

Gentlemen:—Your treasurer would report the following as the state of his account to this date.

| | | |
|---|---|---|
| Dr. | To balance on hand at last settlement, . . . | $183 26 |
| | Received since, . . . . . . . . . . . . . . | 16 00 |
| | Total received, . . . . . . . . | $199 26 |
| Paid janitor per order of society, . . . . . . . | | 5 00 |
| | Balance on hand, . . . . . . . . . . | $194 26 |

He would also report that two of the gentlemen who were elected last year—Drs. Hermon, of Nashville, Tenn., and Evans, of Lexington, Ky., have neglected to pay their initiation fee, so that according to the by-laws, they cannot be considered as members.

Drs. Bonsal, Smith, Talbert and Taft, were appointed to audit the treasurer's account.

*Corresponding Secretary's Report.*—The corresponding secretary would respectfully report, that in accordance with his instructions, the resolutions in regard to the prize essay, were forwarded to and published in the *American Journal of Dental Science*; the *Dental News Letter*; the *Dental Recorder*, and *Dental Register of the West.*

Geo. Watt, *Cor. Sec'y.*

The auditing committee report that "they found upon examination, the report of the treasurer of the M. V. A. correct."

H. R. SMITH,

H. S. TALBERT, } *Committee.*

J. TAFT.

Your committee appointed to have 500 copies of the constitution printed, submit the following report:

Your committee find the constitution needs further emendations, they, therefore, deferred printing until the society could act.

A. M. LESLIE,

C. BONSAL, } *Committee.*

The committee appointed to examine and report on the Essays presented for the prize of $100, by this society, respectfully report that three essays have been presented for their examination, which they have read and of which they present the following: Two of these essays, strictly speaking, fail to meet the requirement of fifty pages duodecimo. Yet they cannot make this a special objection to them, for they would probably make that number of pages, put up in large type, and they feel that the merit of such articles depend more on brevity than length. It appears by the resolutions of the society, that the committee were required to approve and have the best essay printed, that the association may read it also. Yet they were not furnished with the necessary funds for that purpose. This, however, has really not prevented the printing of such as they might have selected; but they have not had the time to make the necessary and careful examinations and get the same printed.

One of these essays the committee would like to have the society examine. If the society sees proper, it can have it printed for the more careful perusal of the association, and thus get their ultimate action on it. The committee think that haste in this matter is to be avoided. The reputation of the society requires that an article which they *endorse* should be such as to commend itself to the profession and public.

The essay which they would like to have the society examine, possesses many excellent points, and is well written, and if it met in a direct manner more especially the prejudices of the community—would be just such as we should desire. We think that a popular treatise should be directed to this special object viz. the correcting the false impressions existing in the community in relation to dental operations. We regret that the original resolutions of the society in relation to essays should not have been more explicit.

JAMES TAYLOR,<br>

J. TAFT,<br>

W. H. GODDARD,<br>

A. M. LESLIE, } *Committee.*

The committee to confer with the College Association reported as follows:

The committee to arrange with the building committee of the College Association, respectfully report, that by arrangements made, space for a cabinet case has been obtained in the cabinet room of the college, and the faculty of the college agree to afford this and the other conveniences required for the society's meetings on such pecuniary consideration as the society may deem proper. Your committee recommend that a suitable cabinet be procured and placed in the allotted space.

A. M. LESLIE,<br>

C. BONSAL,<br>

J. TAYLOR, } *Committee.*

The examining committee reported the name of Dr. C. H. Quinlan, of Chicago, for membership. He was unanimously elected by ballot.

Microscopic committee reported the following: Your committee on microscopic observations, would report, that owing to difficulties which they could not control or overcome in the time allotted to them, they have found it impossible to carry out the design of the appointment, and would respectfully ask further time.

J. TAFT,<br>

JAS. TAYLOR,<br>

A. M. LESLIE, } *Committee.*

Report accepted and time granted.

Dr. Talbert moved that when the meeting adjourns it continues adjourned until 3½ o'clock, P. M., and that the first business then be the president's address. Adopted.

After some discussion touching the report of the committee on essays, Dr. Smith moved, that the reading of the essay therein recommended for examination be the first business of the session in the following morning. Carried.

Dr. Taft moved, that members of the profession not members of the association, be, and are hereby invited, to take part in the discussions if they see proper. Adopted. Adjourned.

*Afternoon Session.*—The minutes of the morning session were read and approved.

The president delivered the opening address. Subject "Association."

Dr. Taft moved, that the thanks of the association be presented to Dr. Goddard for the able address just delivered, and that a copy be requested for publication. Adopted.

The report on dental progress was read by Dr. Leslie, so far as he had time to prepare it. He also presented several specimens, illustrative of improvement; when, on motion, the further reading of the report was deferred until a future session.

Adjourned until 9 A. M., the following day.

*Second Day, Morning Session.*

The minutes of the previous session read and approved.

The examining committee presented the names of Henry Munroe, of Lebanon, Ky., and Jonathan Douthett, of Xenia, Ohio, for membership. Ballot had and gentlemen elected.

On motion, the committee on essays was instructed to report the one which they may think best of those presented. The committee retired and brought in a report, which, on motion, was accepted.

In accordance with instructions of the association, the committee on essays respectfully report that the essay marked No. 3, and presented by "John Gilpin," is in their opinion the best presented, and recommend that the prize promised of $100, be awarded accordingly.

JAMES TAYLOR,
WM. H. GODDARD,
A. M. LESLIE,
J. TAFT.
} *Committee.*

The Essay was then read, and on motion, the recommendation of the committee, awarding the prize ($100) to John Gilpin, author of the best essay, was adopted.

On motion, the envelop containing the true name of the author was opened by the president, when the card of George Watt, D. D. S., M. D., of Xenia, Ohio, was found therein.

On motion, it was resolved, that a committee be appointed to make such amendments in the essay, just read, as may seem necessary, to meet the demands of the profession, and that the author be one of the committee.

On motion, resolved, that the chair appoint the committee. Chair appointed Drs. Watt, Taylor and Taft.

Mr. Goddard presented the following resolution. Whereas, the American Society of Dental Surgeons will hold their next meeting in this city, on the 8th day of May next. Therefore,

*Resolved*, That we deem it expedient that a called meeting of this association be held at that time, and the president is hereby authorized to call such meeting.

*Resolved*, That we cordially invite all members of the profession, not members of this society, to be present, and co-operate with us on the above occasion, that we may be able to extend a hearty welcome and the right hand of fellowship to our eastern brethren. Adopted.

Adjourned to 2½ P. M.

*Afternoon Session.*—Minutes approved.

Dr. Talbert read an essay on the preparation of cavities for filling.

On motion of Dr. Watt, *Resolved*, That the thanks of the association be tendered to Dr. Talbert, and that a copy of his address be requested for publication.

Dr. Smith was granted further time for the preparation of an essay.

Dr. Watt read an essay on alloys of metals.

Dr. Ulrey moved that the thanks of the association be tendered to Dr. Watt, and that his essay be requested for publication. Carried.

Dr. Hamill asked further time for the preparation of his address.

Dr. Ulrey asked to be excused from the consideration of atmospheric plates, which was granted, and he was requested to prepare an essay for our next meeting (annual.)

A motion was made, that the chair appoint a committee to examine extracting instruments, presented in competition for the prize offered last year by the society. Carried.

The chair appointed Drs. Taylor, Talbert and Webster.

Dr. Leslie moved, a committee, consisting of three, be appointed to examine the teeth sent on by Jones, White & Co., and report such action as they may deem proper. The chair appointed Drs. Hamill, Taft and Ulrey.

Dr. Bonsal moved the thanks of the association to Dr. Peebles for his scotch-stone holder.

On motion, resolved, that the business for the morning session, at 9 o'clock, be the election of officers.

The committee on forceps reported, and report accepted.

The committee appointed to examine and report concerning extracting instruments, respectfully report that but one set was placed in their hands. These were presented by Messrs. Sherwood & Brown, and that their adaptation to the teeth, and variety, meeting the wants of the profession in almost all cases, also, finish and style, is such that they recommend them for the award offered.

James Taylor,<br>A. S. Talbert,<br>W. R. Webster, } *Committee.*

On motion, report of committee adopted.

*Resolved*, That an order be drawn on the treasurer, in favor of Messrs. Sherwood & Brown, in accordance with the above.

Mr. Oram, of the firm of Oram & Armstrong, of Philadelphia, presented a card of gum teeth for examination of the society. Referred to committee on porcelain teeth.

Society resumed the regular order of business, discussed Nos. 1 and 2 of miscellaneous business. Adjourned to 7½ o'clock, P. M.

*Evening Session* met and discussed Nos. 3, 4 and 5 of miscellaneous business.

The following report on porcelain teeth was submitted by committee.

Gentlemen of the M. V. A. of D. S. Your committee on artificial teeth, would respectfully report, that the specimens presented by Messrs. Jones, White & McCurdy, are worthy our highest commendation. Every variety of shape, size and color was represented, and we are happy to say that they fully sustain the justly high reputation they already enjoy, among the members of the profession.

We consider those presented to the Miss. Valley Association a valuable acqusition to its cabinet—for which they have our warmest thanks.

Specimens were also examined from the manufactory of Messrs. Oram & Armstrong; these were all gum teeth, and presented a beautiful appearance, and we recommend them to the favorable consideration of the profession.

J. Taft,

J. G. Hamill, } *Committee.*

J. P. Ulrey,

Report accepted and adopted.

*Resolved*, That the business at to-morrow's session, 9½ o'clock, A. M., be election of officers. Adjourned to 8 o'clock A. M.

*Third Day.* Minutes of previous session read and approved.

Dr. Goddard moved, that the thanks of the association be, and are hereby rendered to Messrs. Watt, Sherwood and Brown for the entertainment last evening. Carried.

Society discussed Nos. 6, 7 and 8 of miscellaneous business.

Election of officers followed, with following result:

*President.*—A. S. Talbert.

*Vice-president.*—J. G. Hamill.

*Rec. Secretary.*—A. M. Leslie.

*Cor. Secretary.*—Geo. Watt.

*Treasurer.*—C. Bonsall.

*Executive Com.*—Drs. Smith, Ulrey and Douthett.

*Examining Com.*—Drs. Webster, Taft and Collins.

*Com. on Den. Progress.*—Drs. Watt, Leslie & Smith.

The following gentlemen, in addition to those continued over, were appointed to prepare essays for next annual meeting—W. S. Howe and E. Collins.

The committee on clasps was discharged.

The committee on printing the constitution granted further time.

The president resigned the chair in favor of the president elect—Dr. Talbert.

It was moved, the thanks of the society be tendered to Dr. Goddard, for the able manner he has presided.

On motion of Dr. Taylor, *Resolved,* That the committee on dental progress be requested to continue the report on the subject; embodying all the new and important improvements which have been made for two years past, and ensuing year, and arrange the same for publication by the next annual meeting.

On motion of Dr. Webster, *Resolved,* That the thanks of the association be rendered to Dr. Hunter for his exhibition of specimens, showing the progress made by him in continuous gum work, also, for the communication accompanying the same; and that the association heartily wish him the success he richly merits.

On motion of Dr. Douthett, *Resolved,* That the association request Dr. Taylor—if he deems it practicable—to enlarge the Dental Register, and that we hereby pledge our *influence* to continue and extend its circulation.

On motion of Dr. Taylor, *Resolved,* That this association regard the coviction of Dr. Beale, on the evidence which was presented, as a bad and dangerous precedent, endangering the character and reputation of members of the profession.

Moved an order be drawn on the treasurer for five dollars, in favor of John Reese, the acting janitor, for services done the society.

Dr. Goddard moved, that members send in specimens. Carried.

Dr. Goddard moved, that Dr. Bonsall be appointed to make arrangements for use of rooms for American Society; also, to arrange with the faculty the cost of the accommodations offered this society. Carried.

Dr. Bonsall moved, the secretary be requested to prepare a copy of the minutes in time for publication in the April No. of the Dental Register. Carried.

Minutes read and approved.

On motion, adjournd, to meet again at Dental College, February 20th, 1856, at 10 o'clock, A. M.—*Dental Register.*

### *Special Meeting of the Mississippi Valley Association of Dental Surgeons.*

Special meeting held May 7th to receive the members of the American Society, pursuant to resolution adopted at the annual meeting.

Present—Drs. Taft, Watt, Webster, Goddard, How, Taylor, Smith, McCullom, Brown, Bonsall and Leslie.

The President and Vice-president being absent, on motion of Dr. Goddard, Dr. Bonsall was called to the chair.

On motion, Dr. Watt, chairman, presented the following report:

*To the Mississippi Valley Association:*

Your committee to whom was referred the prize essay, would respectfully report that though unable to act in concert to the extent they desired, yet they have endeavored to carry out the wishes of the association, as well as they could ascertain them from the remarks of members at the last meeting.

The whole essay has been revised and verbal corrections have been made in several places. But our attention was principally directed to the chapters objected to and these are very materially modified, and, as now presented, will, we trust, meet the views of the society. The articles on Extraction, and Mechanical Dentistry are much abridged, and we invite the special attention of the society to them and to the one on "Filing the Teeth." The work is reduced about twelve manuscript pages.

We would therefore recommend the adoption of the following resolution :

*Resolved,* That a committee of three be appointed to publish —— copies of the essay and have the same ready for distribution at the next annual meeting of the association.

Respectfully submitted,

G. WATT,
J. TAYLOR,
J. TAFT.

Report accepted, and on motion, laid over until next day.

Dr. Goddard moved, that when we adjourn, we do so to meet at 8½ o'clock to-morrow. Adopted.

The following communication was presented by the president.

*To the President of the M. V. A. D. S.*

Sir, I hereby resign my place on the committee on "Dental Progress." GEO. WATT.

Dr. Goddard moved to lay on the table until to-morrow. Carried.

On motion adjourned.

*Second day,* 8½ *o'clock, A. M.*

On motion, the following resolutions were unanimously adopted,

*Resolved,* That the members of the Mississippi Valley Association of Dentists and of the profession generally of the West cordially extend to the members of the American Society, and our brethren who are now visiting the city of Cincinnati an invitation to meet us on Wednesday evening, May 9th, at the Walnut Street House to participate with us in a social entertainment.

*Resolved,* That a committee of three be appointed to make all necessary arrangements and extend such invitations as they deem proper, and report as soon as such arrangements are completed.

Dr. Goddard moved that the amended portions of Dr. Watt's essay be now read. Adopted.

*Resolved*, That 300 copies of the essay be published, in the cheapest form, and be distributed by the committee to those members of the profession for amendments and criticism, as they may deem proper, with a request that they will suggest such amendments to the committee, as they may deem advisable, as soon as they can conveniently—said committee to report at our next meeting.

*Resolved*, That the committee on the foregoing essay be instructed to carry it out.

Dr. Goddard moved that the resignation of Dr. Watt be laid on the table indefinitely. Adopted.

By order of the society the name of Dr. E. A. Herman which was left out, by the action of the society at our last meeting, be restored—Dr. J. Taylor having reported that his initiation fee had been paid him previous to our last meeting.

Moved that, when we adjourn, we do so to meet at 4 o'clock, P. M.

Dr. Goddard moved that in the absence of the president, Dr. Taylor be appointed to welcome the American Society in the name of this association, and that Dr. Watt be a committee to invite said society to hold their sessions in this hall.

The members of the American Society being introduced, Dr. Taylor welcomed them to the West and pledged a united effort to make their visit a pleasant one. When on motion the society adjourned.

*Afternoon Session.*—Committee on entertainment reported. Report accepted. Adjourned.

At 9 o'clock, P. M. The society met at the Walnut street Hotel, where, with the members of the American society, together with a number of invited guests, medical and dental, to the number of about fifty, they spent two hours pleasantly around the social board. In answer to toasts, speeches, short but pithy, were made, by Drs. Townsend, Taylor, Dunning, Allen, Jobson and Miller, of the dental profession, and by Professors Wood, Armor and Mendenhall of the medical.

A very pleasant social evening was also enjoyed on the succeeding night at the hospitable board of our worthy ex-president Dr. Chas. Bonsall.

The members of the M. V. Association and others who enjoyed the society of their eastern brethren will recall with pleasure their visit.

A. M. Leslie, *Sec'y.*

---

*The Fifteenth Annual Meeting of the American Society of Dental Surgeons, convened according to Appointment, in the Hall of the Ohio Dental College, in the city of Cincinnati, Ohio, on Tuesday, May* 8*th*, 1855.

Met at 10 o'clock A. M., the President in the chair.

Present Drs. Townsend, Dunning, Wheeler, Miller, Berry, Goddard, Taylor, Allen and Bonsall, together with many other members of the profession; several of them being members of the Mississippi Valley Association of Dental Surgeons, who had met for the purpose of welcoming this society.

In the absence of the Secretary, Dr. Bonsall was chosen Secretary *pro tem.*

On motion, all members of the profession who may be present at any of our sessions, were now invited to participate in our discussions.

The minutes of the last annual meeting were read by the Secretary, and approved, after which the President, Dr. Townsend delivered the opening address, which was listened to with much attention.

Dr. Townsend reported that the chairman of the committee on Microscopic Observations, Dr. J. D. White, had a report to make, but had been unavoidably prevented from attending the meeting; and on motion, the committee was continued to report at the next annual meeting.

On motion of Dr. Taylor a committee of three was appointed to take into consideration, the suggestion made in the President's address; and Drs. Taylor, Dunning and Goddard were appointed and directed to report at the next session.

The chair appointed Drs. Goddard and Taylor to audit the Treasurer's accounts.

Adjourned to meet at 9 o'clock, A. M. to-morrow.

*Morning Session, Second Day.*

Met according to adjournment at 9 o'clock, A. M., with the President in the chair. The minutes of yesterday's meeting were read and approved.

The committee appointed to audit the Treasurer's accounts reported the following:

Your committee appointed to audit the accounts of the Treasurer, would respectfully report, that they have examined the same with the vouchers, and find them correct, and that there is a balance of $227 74 in his hands. All of which is respectfully submitted.

Wm. H. Goddard,<br>James Taylor, } *Committee.*

The next business in order being the report of the committee on the President's address, they offered the following report which was accepted:

*To the American Society, &c.*

Your committee to whom was referred the President's address, would respectfully report:

That they have especially turned their attention to that part of the address which suggests a more liberal, less exclusive, and more national organization: one which shall bring up at our yearly convocations a fair representation from every portion of our extended country.

The committee feel that the profession occupies now a very different position from that which it did when this society was first organized.

That, however admirably adapted the organization was then for the work assigned it, yet having accomplished, as we believe, that work, and been of vast service to the profession—a broader basis for action is now demanded.

The society was organized before we had a dental literature or any effective and well organized system of dental education. In the brief period of its existence, the work of a century has been accomplished, and the impetus, which has wrought such progress, demands a new set of machinery, that we may keep pace with the active spirit of the age.

The committee would therefore recommend that a call be issued by the President of this society, in accordance with the sixth article of the Constitution, "To take into consideration the general subject of associations, and the dissolution of this society; said meeting to be held in Philadelphia, on the day previous to the holding of the meeting called by a number of dentists of that city."

James Taylor,

E. J. Dunning, } *Committee.*

Wm. H. Goddard,

The subject was fully discussed by Drs. Miller, Dunning, Taft, Townsend, Taylor, Leslie, Pease, Watt and Goddard, and the resolutions were unanimously adopted.

On motion of Dr. Goddard the President was requested to give *this afternoon*, to the members who may be present, a description of his manner of plugging teeth.

Dr. Taylor reported the sudden death of Dr. B. N. Freeman, a member of the profession in attendance on the American Society, now in session in this city. Therefore,

*Resolved*, That we deplore the loss thus sustained by the profession, and deeply sympathize with his afflicted family in this sad and unexpected bereavement.

*Resolved*, That, as it was the will of Divine Providence that our friend and brother should die away from his wife and family, deprived of those family comforts so necessary to soothe the passage to the grave, we feel grateful that it was in our power to administer to his wants in the hour of his death. That he died not among strangers but was surrounded by friends.

*Resolved*, That the above be entered on our minutes and that the Secretary transmit a copy of the same to the family of the deceased.

James Taylor,
Wm. H. Goddard,
E. D. Wheeler.

On motion adjourned to meet at 3½ o'clock, P. M.

*Afternoon Session.*—Met according to adjournment at 3½ o'clock, P. M., when the minutes of the morning session were read and approved.

The President spent some time, apparently very much to the satisfaction of members, in detailing his method of plugging various kinds of cavities in teeth, and under many different circumstances, after which there was a general discussion on the subject of plugging, participated in by Drs. Miller, Bonsall, Taylor, Wheeler and others.

Adjourned till 9 o'clock, A. M., to-morrow.

*Third Day, Morning Session.*

Met at 9 o'clock, A. M., according to adjournment. The minutes of last session were read and adopted.

On motion of Mr. Goddard the election of officers was made the special order of business for 11 o'clock this morning.

On motion, Dr. Townsend was requested to read to the society such portions of two lectures delivered by him to the dental class in the Philadelphia College, as he may think proper.

This request was complied with, after which it was, on motion of Dr. Taylor,

*Resolved*, That the lectures read by our President this day on professional courtesy, &c., be requested for publication in the "Dental Register."

Drs. Goddard and Dunning being appointed as a nominating committee, reported, that under the circumstances it was thought best to continue the present board of officers in office until the next meeting—said report was accepted and unanimously approved.

Dr. Dunning reported, having found accidentally a much superior sharpening and polishing stone to the Arkansas stone; it is called Missouri oil stone.

The subject of separating the bicuspid teeth, with the best form of space to be left, was now spoken to by Drs. Dunning, Miller, Bishop, Webster and others.

Adjourned to meet at 3 o'clock, P. M.

*Afternoon Session.*—Met at 3 o'clock, P. M., when the subject of removing the first or second bicuspid or first molar, for the purpose of giving room, where there was a prospect of a crowded dentition, was now discussed by Drs. Dunning, Bonsall, Goddard, Taylor, Robbins, Smith, Wheeler, Miller, Bishop, Webster and others.

On motion the treasurer was directed to pay the janitor of the Ohio Dental College $8 for services.

Adjourned to meet at the residence of Dr. Bonsall, at 8 o'clock this evening.

*Evening Session.*—Met according to adjournment at the residence of Dr. Bonsall, together with several members of the medical profession, and others, and held a general *tooth talk.*

On motion of Dr. Dunning, it was

*Resolved,* That the thanks of the American Society of Dental Surgeons be, and they hereby are tendered to our brethren of the Mississippi Valley Association, and to the officers of the Ohio College of Dental Surgery for the courtesy and kindness extended to us during our sessions in Cincinnati.

On motion of Dr. Bonsall,

*Resolved,* That the thanks of this society be tendered to the President, Dr. Townsend, for the able, efficient and impartial manner in which he has filled the chair during our sessions.

On motion, Drs. Bonsall and Taylor were appointed a committee to publish the proceedings of the meeting.

Adjourned *sine die.*

CHARLES BONSALL,

*Corresponding and Recording Secretary pro tem.*

[*Dental Register.*

